# Deaths in Cottage Hospitals

**Published:** 1918-04-27

**Authors:** Charles A. Bailey

**Affiliations:** Vice-Chairman, Buxton Cottage Hospital. Overstone, Robertson Road, Buxton, April 16, 1918.


### THE EDITOR'S LETTER-BOX.

### Deaths in Cottage Hospitals.

*To the Editor of* The Hospital.

Sir,—My attention has been called to the paragraph in your issue of the 13th inst., relating to the deaths in the Buxton and District Cottage Hospital. It is most unfortunate that through a printer's error in the report of the annual meeting published in one of our local papers much misapprehension has been caused. The facts stated at the meeting (as will be seen from the *Buxton Herald* of April 4) were, that out of 988 cases admitted there had only been thirty-eight deaths. This, for the period of five and a half years since the hospital was opened, was an average of about seven per year, with a percentage of less than four.

If you will kindly insert this explanation in your next issue the committee will be very grateful.

Yours faithfully,

Charles A. Bailey,

Vice-Chairman, Buxton Cottage Hospital.

Overstone, Robertson Road, Buxton, April 16, 1918.

